# Proteomic Profiling Identifies Predictive Signatures for Progression Risk in Patients with Advanced-Stage Follicular Lymphoma

**DOI:** 10.3390/cancers16193278

**Published:** 2024-09-26

**Authors:** Jonas Klejs Hemmingsen, Marie Hairing Enemark, Emma Frasez Sørensen, Kristina Lystlund Lauridsen, Stephen Jacques Hamilton-Dutoit, Robert Kridel, Bent Honoré, Maja Ludvigsen

**Affiliations:** 1 Department of Hematology, Aarhus University Hospital, 8200 Aarhus, Denmark; johemm@rm.dk (J.K.H.); mariem@rm.dk (M.H.E.); emmase@rm.dk (E.F.S.); 2 Department of Clinical Medicine, Aarhus University, 8000 Aarhus, Denmark; 3 Department of Pathology, Aarhus University Hospital, 8200 Aarhus, Denmark; krislaur@rm.dk (K.L.L.); stephami@rm.dk (S.J.H.-D.); 4 Princess Margaret Cancer Centre—University Health Network, Toronto, ON M5G 2C4, Canada; robert.kridel@uhn.ca; 5 Department of Biomedicine, Aarhus University, 8000 Aarhus, Denmark

**Keywords:** non-Hodgkin’s lymphoma, follicular lymphoma, progression, proteomics, risk stratification

## Abstract

**Simple Summary:**

This research aims to identify new protein markers that can help predict the risk of disease progression in patients with follicular lymphoma (FL) at the time of diagnosis. By analyzing a large number of proteins in FL samples, the study found significant differences in protein composition among patients, which may help distinguish those at higher risk of disease progression. These findings could improve our understanding of FL biology and lead to the discovery of new biomarkers or treatment targets, potentially allowing for more personalized and effective treatment strategies for FL patients, improving their outcomes.

**Abstract:**

**Background:** Follicular lymphoma (FL) is characterized by an indolent nature and generally favorable prognosis, yet poses a particular clinical challenge, since disease progression is observed in a notable subset of patients. Currently, it is not possible to anticipate which patients will be at risk of progression, highlighting the need for reliable predictive biomarkers that can be detected early in the disease. **Methods:** We applied tandem-mass-tag labelled nano-liquid chromatography tandem mass spectrometry (nLC-MS/MS) on 48 diagnostic formalin-fixed, paraffin-embedded tumor samples from patients with advanced-stage FL. Of these, 17 experienced subsequent progression (subsequently-progressing, sp-FL) while 31 did not (non-progressing, np-FL). **Results:** We identified 99 proteins that were significantly differentially expressed between sp-FL samples and np-FL samples (*p* < 0.05; log_2_-fold changes between 0.2 and −1.3). Based on this subset of proteins, we classified patients into high-risk and low-risk subgroups using unsupervised machine learning techniques. Pathway analyses of the identified proteins revealed aberrancies within the immune system and cellular energy metabolism. In addition, two proteins were selected for immunohistochemical evaluation, namely stimulator of interferon genes 1 (STING1) and isocitrate dehydrogenase 2 (IDH2). Notably, IDH2 retained significantly lower expression levels in sp-FL samples compared with np-FL samples (*p* = 0.034). Low IDH2 expression correlated with shorter progression-free survival (PFS, *p* = 0.020). **Conclusions:** This study provides evidence for some of the biological mechanisms likely to be involved in FL progression and, importantly, identifies potential predictive biomarkers for improvement of risk stratification up-front at time of FL diagnosis.

## 1. Introduction

Follicular lymphoma (FL) is the second most common non-Hodgkin’s lymphoma entity in Western countries. The disease is generally characterized by indolent growth, long responses to first-line therapy, and favorable outcomes with a median overall survival (OS) exceeding 10 years [1,2]. Approximately 90% of patients with FL present with advanced-stage III-IV disease, which largely determines the choice of treatment [1]. With the introduction of immunochemotherapy, advanced-stage FL patients are now experiencing 5-year OS rates of more than 90% [3]. However, a small yet important subset of patients (15–20%) experience a more aggressive disease course with early progression of disease, which includes treatment refractoriness, histological transformation (HT), and/or early death [4,5,6,7,8,9,10]. Besides early events (i.e., within 24 months of initial diagnosis or treatment initiation), it is estimated that approximately 25–35% will experience histological transformation to a more aggressive subtype, often diffuse large B-cell lymphoma [2]. Thus, FL is generally considered a heterogenetic entity reflected both in disease trajectory of the patients as well as the biology of the disease [2]. Substantial efforts have been made to identify prognostic factors, both clinical and biological, to predict the course of the disease and patient outcomes already at the time of diagnosis [11,12,13,14,15,16,17,18]. Despite these efforts, the search for reliable biomarkers and risk models that consistently predict patient outcomes continues [13].

In this context, our group has used proteomics to try to identify predictive biomarkers in several types of lymphoma [19,20,21,22]. The large-scale study of proteins is crucial, as proteins represent the functional output of genetic information and are directly involved in cellular processes and disease pathology [23]. Therefore, gaining a deeper understanding of the proteome in FL could reveal crucial insights into the biological mechanisms driving the advancement of the disease and potentially identify biomarkers associated with its progression.

In this study, we employed high-throughput mass spectrometry (MS) to analyze the protein profiles of diagnostic lymphoma tissue, in the context of high-risk disease with progression as the endpoint.

This approach allowed us to identify proteins that were differentially expressed among tumor samples from patients who experienced progression and those who did not.

## 2. Patients and Methods

All analyses were performed using primary, diagnostic, formalin-fixed, paraffin-embedded (FFPE) tumor samples from a cohort consisting of 49 FL patients diagnosed with advanced-stage (III–IV) FL grade 1–3A at Aarhus University Hospital between 2003 and 2014. All patients were treated with immunochemotherapy. One sample was excluded from the cohort because of technical issues, leaving 48 samples for analysis (Supplementary Figure S1). These included 31 FL patients who did not experience disease progression for at least 5 years after treatment initiation (non-progressing FL, np-FL) and 17 patients with a histologically confirmed subsequent progression (subsequently progressing FL, sp-FL). We defined the time of progression as the date that the second line of therapy was initiated. Samples from diagnosis and progression were histologically reviewed by an experienced hematopathologist according to the 2017 WHO criteria [2]. Clinical data from all patients were collected from the Danish Lymphoma Registry (Table 1) [24]. Patients were meticulously selected based on several criteria: grade 1, 2, or 3A, no composite histology (i.e., no component of large B-cell lymphomas), no prior treatment, advanced-stage (Ann Arbor III-IV and/or bulk > 10 cm and/or B-symptoms), and an available FLIPI risk score. These criteria ensured a well-defined cohort. The study period was from 2003 until the last follow-up in 2023. Patients who experienced progression were followed from initial diagnosis until progression date; those who did not were followed from the date of diagnosis until the last date of follow-up, with the exception of one patient who died 54 months after the initial diagnosis (Supplementary Figure S2). Consequently, the minimum follow-up duration was 9 years for all cases except one. The study was approved by the National Committee on Health Research Ethics in Denmark (2007991) and the Danish Data Protection Agency (1-16-02-237-20) and conducted in compliance with the principles of the Helsinki Declaration.

### 2.1. Identification of Differentially Expressed Proteins

To identify differentially expressed proteins, a tandem mass tag (TMT) labelled nano-liquid chromatography tandem MS (nLC-MS/MS)-based proteomic analysis was performed. Bioinformatic analysis was performed using the Search Tool for the Retrieval of Interacting Genes/Proteins (STRING) database [25]. Expression levels of two selected proteins, stimulator of interferon genes 1 (STING1) and isocitrate dehydrogenase 2 (IDH2), identified by proteomics, were further evaluated in the same cohort using immunohistochemistry (IHC). IHC staining was quantified by digital image analysis, the quantified staining being expressed as area fractions (AFs), calculated as the ratio of the stained area to the overall area within the region of interest. A detailed description of the MS-based proteomics and IHC staining protocols is given in the Supplementary Methods and Supplementary Table S6.

### 2.2. Statistical Analysis

Differences in clinicopathological features were assessed using either a χ^2^-test or Fisher’s exact test as appropriate. For evaluating fold changes in differentially expressed proteins between np-FL and sp-FL samples a student’s *t*-test was employed on the average expression levels of the proteins in each group. Only proteins without missing values were subjected to principal component analyses (PCAs) to avoid the need for data imputation. Hierarchical clustering was performed with Euclidean distance for measuring dissimilarity and Ward’s method for cluster linkage. The differences in AFs between np-FL and sp-FL samples were analyzed using an independent Mann–Whitney U test. Spearman’s rank test was used to explore the correlation between biomarker expression and clinicopathological features. To determine high vs. low biomarker expression and for time-dependent outcomes, cutoff values were established using receiver operating characteristic curve analysis, with the optimal cutoff point calculated from the Youden’s index. Time-dependent outcomes were examined through Kaplan–Meier and log-rank methods, with progression-free survival (PFS) as the primary endpoint. PFS was defined from the initial FL diagnosis to time of progression. Statistical significance was set at *p* < 0.05. All statistical analyses were conducted using RStudio (version 4.25).

## 3. Results

### 3.1. Patient Characteristics

Clinicopathological characteristics of the 48 included advanced-stage FL patients are summarized in Table 1. The cohort consisted of 26 males and 22 females, with ages at diagnosis ranging from 30 to 82 years and a median age of 59 years. Out of these, 17 patients experienced disease progression, while 31 did not. There were significantly more patients younger than 60 years in the sp-FL group compared with the np-FL group (*p* = 0.034). Otherwise, there were no differences between the two groups in the studied clinicopathological features. The median time to progression was 4.03 years (range 0.32–7.65 years).

### 3.2. Proteomic Profiling Reveals Differentially Expressed Proteins

**Figure 1:**
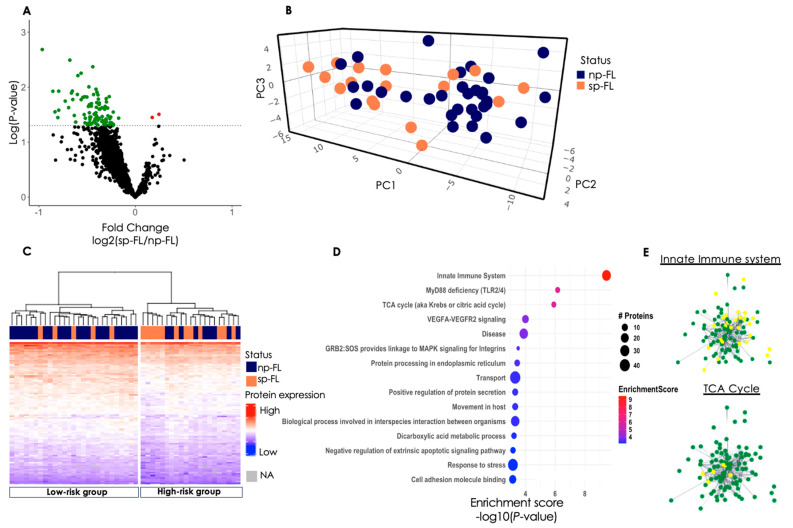
Identified proteomic differences between sp-FL and np-FL samples. (**A**) Volcano plot of all 1940 identified proteins. The x-axis shows the log_2_-fold change of the subsequently progressing group versus the non-progressing group, and the y-axis shows the −log(*p*-value). Significantly differentially expressed proteins are color-coded according to whether they are upregulated (red) or downregulated (green) in subsequently progressing samples. The dotted line indicates the significance level of *p* < 0.05. (**B**) 3D PCA plot of significantly differentially expressed proteins between sp-FL and np-FL samples. Here, 93 proteins identified from *p* < 0.05 (without missing values) were used. (**C**) Heatmap and hierarchical clustering of the 99 identified proteins. Each row represents one protein, and each column represents one sample. (**D**) Enrichment analysis plot based on the 99 significantly differentially expressed proteins. The biological processes/pathways are seen to the left. The dot size correlates to the number of involved proteins where the color corresponds to the enrichment score. (**E**) Protein-Protein interaction network of the 99 proteins identified. Nodes represent proteins and edges visualize interactions, either functional or physical. Yellow indicates proteins involved in said pathway; green, protein not involved in the pathway. Abbreviations: NA, not assigned; np-FL, non-progressing follicular lymphoma; PC, principal component; sp-FL, subsequently progressing follicular lymphoma; TCA, tricarboxylic acid cycle.

From the nLC-MS/MS a total of 1940 proteins were identified across 48 samples. PCA based on all identified proteins did not reveal any noticeable clustering of the samples (Supplementary Figure S3). Among the identified proteins, 99 proteins were significantly differentially expressed (*p* < 0.05) between np-FL and sp-FL samples. Surprisingly, we observed a general picture of protein expression being downregulated in sp-FL samples, with 97 of the 99 significantly differentially expressed proteins being downregulated (log_2_ fold changes −0.17 to −1.28) and only two upregulated (log_2_ fold changes 0.17 to 0.24) sp-FL (Figure 1A; Supplementary Table S1). Among the 97 downregulated proteins were several immune-related proteins, including STING1, CD14, and complement receptor 2 (CR2), as well as proteins involved in cellular respiratory mechanisms, including IDH2, IDH3A, and dihydrolipoyl dehydrogenase (DLD).

Next, to determine whether the different protein-expression profiles could differentiate FL samples according to risk of subsequent progression, unsupervised PCA was performed with input of significantly differentially expressed proteins. This revealed a pattern showing the majority of samples clustering, corresponding to either np-FL or sp-FL samples, respectively, with few remaining samples intermingled, reflecting possible risk subgroups of FL tumors with progression as the endpoint (Figure 1B). The same tendency was observed when performing hierarchical clustering based on the 99 significantly differentially expressed proteins between sp-FL and np-FL samples. Here, samples were clustered into two major groups; one group was designated as a low-risk group (LRG; 22 np-FL and five sp-FL samples) and one group as a high-risk group (HRG; nine np-FL and 12 sp-FL samples) (Figure 1C). Samples within these two groups were then reanalyzed separately with the aim of identifying candidate proteins with even higher potential for predicting subsequent progression.

#### 3.2.1. Low-Risk Group Analysis

**Figure 2:**
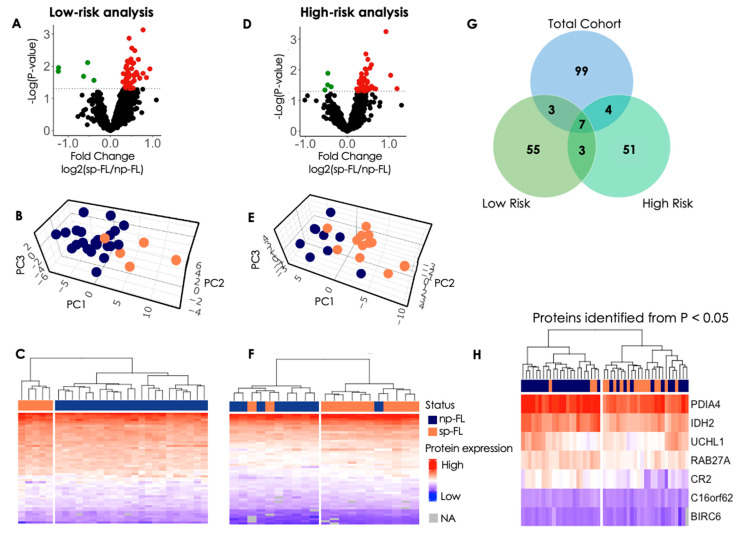
Re-analysis of low-risk and high-risk subgroups. (**A**) Volcano plot of all 1940 identified proteins. The x-axis shows the log_2_-fold change of the subsequently progressing group versus the non-progressing group, and the y-axis shows the −log(*p*-value). Significantly differentially expressed proteins are color-coded according to whether they are upregulated (red) or downregulated (green) in subsequently progressing samples. (**B**) 3D PCA plot of significantly differentially expressed proteins between sp-FL and np-FL samples. Here, 54 proteins identified from *p* < 0.05 (without missing values) were used. (**C**) Heatmap and hierarchical clustering of the 55 identified proteins. Each row represents one protein, and each column represents one patient. (**D**) Volcano plot of all 1940 identified proteins. The x-axis shows the log_2_-fold change of the subsequently progressing group versus the non-progressing group, and the y-axis shows the −log(*p*-value). Significantly differentially expressed proteins are color-coded according to whether they are upregulated (red) or downregulated (green) in subsequently progressing samples. (**E**) 3D PCA plot of significantly differentially expressed proteins between sp-FL and np-FL samples. Here, 42 proteins identified from *p* < 0.05 (without missing values) were used. (**F**) Heatmap and hierarchical clustering of the 51 identified proteins. Each row represents one protein, and each column represents one patient. (**G**) Venn diagram showing the number of differentially expressed proteins from each analysis and common proteins between the analyses. Three proteins were common between low risk and the total cohort, while four were common between high risk and the total cohort. In total, seven proteins were common between all three analyses, as shown in the diagram. (**H**) The seven proteins in common between all analyses used for hierarchical clustering in the entire cohort. Abbreviations: NA, not assigned; np-FL, non-progressing follicular lymphoma; PC, principal component; sp-FL, subsequently-progressing follicular lymphoma.

In the investigation of the LRG, 22 np-FL and five sp-FL samples underwent reanalysis. Here, 55 proteins were identified as significantly differentially expressed (*p* < 0.05) between the np-FL and sp-FL samples within the LRG (Supplementary Table S2). Notably, in this group only five proteins were downregulated in the sp-FL samples while 50 were upregulated (Figure 2A). Interestingly, the protein expression exhibited greater symmetry between sp-FL and np-FL samples, suggesting a more balanced distribution of protein levels across the groups. With input of the 55 significantly differentially expressed proteins, PCA were able to distinguish np-FL and sp-FL samples in the LRG with a clear separation (Figure 2B). This separation was underpinned by hierarchical clustering, where the inclusion of significantly differentially expressed proteins revealed a distinct separation into clusters comprising all five sp-FL samples and a cluster encompassing all 22 np-FL samples (Figure 2C).

#### 3.2.2. High-Risk Group Analysis

For the analysis of the HRG, nine np-FL and 12 sp-FL samples were reanalyzed. Here, 51 proteins were identified as significantly differentially expressed (*p* < 0.05) between the np-FL and sp-FL high-risk samples (Supplementary Table S3). Interestingly, once again only four of these proteins were downregulated in sp-FL samples while 47 were upregulated, and the general protein-expression level between them was more similar (Figure 2D). PCA with the input of the 51 significantly differentially expressed proteins was able to further discriminate np-FL and sp-FL samples within the HRG, revealing a separation with only a few samples remaining intermingled (Figure 2E). Accordingly, with input of the 51 significantly differentially expressed proteins, the hierarchical clustering analyses revealed two clusters, one cluster consisting of one np-FL and 10 sp-FL samples, and another cluster containing two sp-FL samples intermingled with the remaining eight np-FL samples (Figure 2F). Clinicopathological data showed no distinct explanation for the placement of the one np-FL sample and two sp-FL samples, respectively. However, the two sp-FL samples were both from female patients older than 60 years of age with an Ann Arbor stage III. One of the two progressed within 0.4 years, whereas the other progressed after 6 years. In contrast, the one np-FL sample intermingled with the 10 sp-FL samples was younger than 60 years of age at diagnosis and had an Ann Arbor stage of IV.

#### 3.2.3. Insights from Protein Profiles in High- and Low-Risk Groups

From the analyses described above, two different subsets of differential protein profiles (HRG, *n* = 55; LRG, *n* = 51) were identified, which were both able to enhance the separation of np-FL and sp-FL samples in their respective analyses. Therefore, to evaluate how these proteins would perform on the total cohort, hierarchical clustering was performed with the input of differentially expressed proteins identified from either LRG or HRG analyses, respectively. However, none of these two subsets performed better than the 99 differentially expressed proteins identified in the first analysis. (Supplementary Figure S4A,B). This was perhaps not surprising, since only seven proteins were common in all three analyses (Figure 2G). Interestingly, hierarchical clustering based on only these seven proteins revealed an improved separation of the two groups compared with the initial 99 proteins, as only three sp-FL remained intermingled within the np-FL samples (Figure 2H). Notably, the seven proteins retained the same tendency in the LRG, HRG, and initial analysis. Clinicopathological data showed no distinct explanation for the placement of these three sp-FL samples. However, all three sp-FL patients experienced progression after more than 6 years, implying late progression; thus, the protein-expression profile at FL diagnosis may mirror np-FL more than sp-FL with progression disease at an earlier time point.

#### 3.2.4. Disturbed Cellular Pathways at FL Diagnosis 

To investigate the possible biological impact of differences in protein expression on important cellular pathways and processes associated with disease progression, a gene-enrichment analysis of the 99 significantly differentially expressed proteins was computed via the STRING database. The protein network consisted of 99 nodes and 735 edges (*p* < 0.001). Within this network, different clusters of proteins were observed among the disturbed pathways, with several proteins being involved in multiple processes, suggesting a multifaceted role for these proteins in orchestrating various cellular functions. (Supplementary Table S4). The most noticeable changes were observed in the processes of the innate immune system, MyD88 deficiency (TLR2/4), and the tricarboxylic acid (TCA) cycle (Figure 1D,E). The proteins involved in the MyD88 deficiency were also found in the innate immune system. Thus, we focused solely on the innate immune system and the TCA cycle.

### 3.3. Immunohistochemical Evaluation of Selected Proteins Identifies Markers Capable of Predicting Progression

In the large-scale MS-based study, numerous proteins associated with the innate immune system and the TCA cycle were identified. Interestingly, the STING1 protein exhibiting significant differential expression in the proteomic analysis is associated with the innate immune system, whereas the IDH2 protein is related to the TCA cycle. Both proteins were further evaluated by immunohistochemistry in the tumor tissues. 

**Figure 3:**
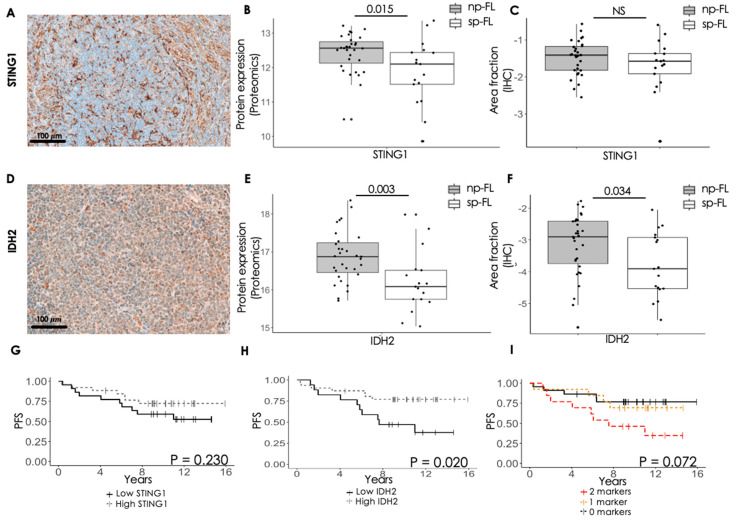
Immunohistochemical evaluation of STING1 and IDH2. (**A**) Representative image of STING1 staining with 100 μm line scale bar (20× magnification). (**B**) Boxplot of STING1 protein expression obtained from MS-based proteomics data. (**C**) Boxplot of STING1 protein expression obtained from immunohistochemical staining (*p* = 0.381). The y-axis is log-scaled. (**D**) Representative image of IDH2 staining with 100 μm line scale bar (20× magnification). (**E**) Boxplot of IDH2 protein expression obtained from MS-based proteomics data. (**F**) Boxplot of IDH2 protein expression obtained from immunohistochemical staining. The y-axis is log-scaled. (**G**) Association between STING1 expression and PFS (cutoff, AF = 0.2132). (**H**) Association between IDH2 expression and PFS (cutoff, AF = 0.021). (**I**) Association between PFS and combined low expression of 0, 1, or 2 of the analyzed markers. Abbreviations: np-FL, non-progressing follicular lymphoma; PFS, progression-free survival; sp-FL, subsequently progressing follicular lymphoma.

STING1 showed very strong expression within the cytoplasm of the cells, with most of the STING1-expressing cells located on the rim of the follicles (Figure 3A and Supplementary Figure S5A,B). IDH2 showed a generally strong and diffuse cytoplasmatic staining, located in both intra- and interfollicular areas (Figure 3D and Supplementary Figure S5C,D). When evaluating the staining, STING1 showed no difference in expression level between sp-FL and np-FL (*p* = 0.481), inconsistent with the MS proteomic analysis (Figure 3B,C). On the other hand, IDH2 revealed significantly lower expression levels in the sp-FL samples (*p* = 0.034), which was consistent with the finding in the MS proteomic analyses (Figure 3E,F).

Interestingly, expression levels of the two markers showed a significantly moderate positive correlation with each other (*p* < 0.001, ρ = 0.47; Supplementary Table S5). Moreover, STING1 showed significantly weak positive correlation to age (*p* = 0.014, ρ = 0.35) as well as a significantly weak negative correlation to samples from patients with ≥4 nodal sites involved. In addition, STING1 showed a tendency towards a negative weak correlation to patients with progression of disease within 24 months (POD24) (*p* = 0.093, ρ = −0.25). IDH2 showed a significantly weak positive correlation to FL grade (*p* = 0.031, ρ = 0.32), a significantly weak negative correlation to B-symptoms (*p* = 0.018, ρ = −0.34), and a significantly weak negative correlation with progression of disease (*p* = 0.032, ρ = −0.31). In addition, IDH2 showed a tendency towards a negative weak correlation to samples from patients with ≥4 nodal sites involved (*p* = 0.065, ρ = −0.27).

Low protein-expression level of STING1 at the time of initial FL diagnosis showed no correlation with PFS (*p* = 0.230), while low expression level of IDH2 was associated with significantly shorter PFS (*p* = 0.020) (Figure 3G,H). Interestingly, combining the expression levels of both markers, low expression of both STING1 and IDH2 did not retains its significance (*p* = 0.072), yet a trending association between samples with two markers and inferior PFS was observed (Figure 3I). This indicates that alterations within the expression of IDH2 and perhaps the TCA cycle might be involved in disease progression. In contrast, the indication that the innate immune system might be involved remains unclear, warranting further study.

## 4. Discussion

Using high-throughput proteomics, we demonstrated that diagnostic tumor samples from FL patients exhibit distinct protein-expression patterns associated with the risk of disease progression. Furthermore, our research pinpointed potential proteins worthy of consideration as candidate prognostic biomarkers. The comparison of protein profiles of sp-FL and np-FL samples revealed important differences between groups, which at the time of diagnosis showed similar clinicopathological features that would normally preclude the possibility for upfront risk stratification. Here, we showed that the two subgroups (sp-FL and np-FL) showed significant differences in their protein-expression profiles despite the groups having similar disease histology (grade 1–3A). To date, the processes driving progression in FL remain unresolved, although studies have proposed different hypotheses to explain what mechanisms might contribute to the progression of FL disease, namely individual genetic alterations, genomics properties, and the tumor microenvironment (TME) [7,9,26]. Our study advances our understanding of FL progression, providing support for these concepts at the protein level.

Notably, we identified a network of proteins centering around the innate immune system, including STING1, CD14, and CR2, all being downregulated in sp-FL samples compared with np-FL samples. Upon cyclic GMP-AMP synthase recognizing foreign DNA, STING1 is activated by sensing the second messenger cyclic GMP-AMP, initiating an interferon type I (IFN-I) response [27]. The release of IFN-I has the capacity to stimulate and activate immune cell subsets (e.g., natural killer cells), thereby augmenting their capability to target and eliminate tumor cells [28]. A study conducted by Lekh et al. investigated the efficacy of STING agonists (STINGa) in reprogramming macrophages and enhancing antibody immunotherapy, both in vitro and in vivo. Their findings indicated that STINGa treatment led to the improvement of antibody immunotherapy by modulating the tumor microenvironment [29]. Thus, although our combined analyses of MS-based proteomics and immunohistochemical evaluation failed to confirm whether STING1 has a direct role in the progression of FL, our results warrant further investigation of STING1’s role in disease progression.

Interestingly, we found downregulation of the monocyte/macrophage marker CD14 in sp-FL samples, which could indicate a lower number of macrophages present in the tissue [30]. STING1 is expressed in many cell types, including macrophages. Thus, the observed low STING1 expression in the proteomic assessment could be a surrogate marker for low macrophages infiltration of the TME [31]. Nonetheless, these results imply a potentially immunosuppressive TME in diagnostic tumor samples from sp-FL patients. This is consistent with findings from a previous study by Tobin et al., in which they observed that FL biopsies were characterized by a lower presence of macrophages and by fewer T-cell clone populations, defined as low immune infiltration. Importantly, they found that patients with tumors demonstrating low immune infiltration were enriched for progression events [9].

Our study also identified downregulated proteins involved in the TCA cycle, a metabolic pathway commonly disturbed in cancers [32]. These include IDH2 and IDH3A, both part of the IDH family responsible for the conversion of isocitrate to α-ketogluterate [33]. Dysregulation of proteins involved in the TCA cycle, can potentially play a role in progression through various mechanisms, such as alterations in cellular redox homeostasis, increasing oxidative stress and thus promotion of DNA damage [34]. Furthermore, mutations in *IDH* genes, particularly *IDH2*, have been shown to result in the production of oncometabolites, such as 2-hydroxyglutarate, which can disrupt cellular signaling pathways and epigenetic regulation, thereby contributing to tumor initiation and progression [35,36]. Moreover, it is well established that cancer cells need to undergo metabolic reprogramming to adapt to the unfavorable conditions in the TME [37,38]. In angioimmunoblastic T-cell lymphomas, mutations in the *IDH* genes are well-known; thus, further investigations of the *IDH* family might focus on the mutational status to understand better the presumed pathogenic association of *IDH* in FL progression [39,40]. In our study, we found a significantly lower IDH2 expression in sp-FL tumors compared with np-FL tumors, which is in contrast to many studies which have shown that overexpression of proteins in the IDH family supports cancer growth [41,42,43,44]. We observed a general downregulation in proteins involved in the TCA cycle, and the lowered IDH2 expression perhaps represents an altered TCA cycle that potentially is associated with progression of FL disease.

Importantly, based on the expression levels of identified markers, we were able to identify patients with inferior PFS, based on IDH2 expression levels. This was not the case for STING1. Notably, the two markers showed a positive correlation with each other. However, the PFS did not retain its significance when combining the two markers. Nonetheless, our data suggests that the innate immune system and the TCA cycle may be associated with the risk of progression in patients with advanced-stage FL. Further studies in larger, independent patient cohorts are warranted to investigate whether these two pathways and their respective markers are of prognostic or predictive value for progression in advanced-stage FL. Furthermore, we identified additional networks of proteins centering other pathways related to cancer that merit further study.

There have been few published studies that have used MS-based proteomic characterization of FL and FL progression. Our group recently performed label-free quantification MS-based proteomics on a cohort of paired FL samples to investigate histological transformation [19]. From this study, we identified a network of proteins involved in apoptotic signaling, among these caspase 3 (CASP3). In the present study, CASP3 was not identified; however, another member of the caspase gene family, caspase 4 (CASP4), did show significant differences between sp-FL and np-FL samples (*p* = 0.038), implicating apoptotic regulation in FL disease progression as well [45]. Similarly, other studies have similarly investigated the apoptotic signaling pathway within FL, and consistently observed disturbances among proteins involved in apoptosis [46,47,48,49,50]. This suggests the need for further study into the role of apoptosis-related proteins as potential markers for progression of disease within FL.

In a recent study, Deng et al. employed TMT-labelled MS-based proteomics to investigate mechanisms of early progression within FL. They discovered heightened expression of GLUT1 in samples from FL patients experiencing POD24 compared to those in long-term remission. Additionally, they observed upregulation of immunosuppressive markers and downregulation of inflammatory cytokines [51]. These findings agree with our presented results, suggesting a role of the immune system and metabolic processes within disease progression of FL.

This large-scale proteomic study was performed as a hypothesis-generating investigation, with the aim of identifying novel markers important for progression in FL. When evaluating STING1 with immunohistochemical staining, no significant difference was observed between sp-FL and np-FL samples, in contrast to our findings using MS-based proteomics. Several factors may underlie this inconsistency, such as differences in sensitivity and specificity between MS and IHC as well as variability in sample-preparation techniques. Importantly, MS relies on quantification of reporter ions labelled peptides derived from different proteoforms belonging to protein groups, whereas IHC is based on antibodies that recognize specific epitopes in proteins. Thus, the two methods might quantify different sets of proteins within the protein group. Additionally, the proteomic analysis was performed on whole tissue sections, thus including both neoplastic and non-neoplastic, and both lymphoid and non-lymphoid TMEs. In the present study, MS-based proteomics is used as a discovery method and thus, relevant proteins were further investigated with a clinically implementable method—IHC. We investigated the proteins as they reflect the active part of the tumor; however, because of the heterogeneity among patients, pinpointing specific proteins can be challenging.

To circumvent this limitation, immunohistochemical staining was performed. Besides constituting a clinically applicable method, it allows for investigation of the protein expression in the malignant cells and their surroundings, getting a perspective on both the tumor cells and microenvironmental cells. Thus, the staining quantification in the immunohistochemical analyses was confined to the region of interest, thereby evaluating the neoplastic cells and the surrounding TME, restricted to lymphoid tissue of the biopsies. Investigations in independent and larger cohorts are warranted to elucidate the possible importance of these methodological differences.

Progression of FL disease has in the recent years become an established clinically relevant endpoint [5]. However, to fully benefit from this endpoint, anticipating patients at high risk and low risk at time of diagnosis is necessary. Thus, the study only included FL patients rather than healthy individuals, as the aim was to describe protein-expression differences among FL patients with or without subsequent progression.

We presented data from a comprehensive study of proteins that underlie the biological differences observed among individual FL tumors. These data may predict downstream effects and identify new biomarkers with predictive or prognostic value for disease progression, or even new targets for potential future treatments.

## 5. Conclusions

Large-scale proteomic analysis revealed significant variations in protein compositions within diagnostic FL samples, facilitating separation of FL patients based on their risk of subsequent progression. Our findings highlight a novel set of proteins exhibiting differential expression, notably implicated in the innate immune system and the cellular energy metabolism, offering promise in predicting the risk of progression at the time of FL diagnosis. At present, FL patients experience diverse clinical outcomes, with disease progression being a significant endpoint. The capacity to reliably identify patients at FL diagnosis who are at increased risk of progression would be an important clinical breakthrough.

## Figures and Tables

**Table 1:** Patients’ clinicopathological features.

	Total, *n* = 48 *n* (%)	sp-FL, *n* = 17 *n* (%)	np-FL, *n* = 31 *n* (%)	*p*-Value
*Sex*				NS
Male	26 (54)	10 (59)	16 (52)	
Female	22 (46)	7 (41)	15 (48)	
*Age at diagnosis, y*				NS
Median	59	56	60	
Range	30–82	30–78	39–82	
*Age ≤ 60*				0.034
No	26 (54)	13 (76)	13 (42)	
Yes	22 (46)	4 (24)	18 (58)	
*Site of Biopsy*				NS
Lymph node	45 (94)	17 (100)	28 (91)	
Gl. parotidea	1 (2)	0 (0)	1 (3)	
Ileum	1 (2)	0 (0)	1 (3)	
Nasal cavity	1 (2)	0 (0)	1 (3)	
*FL grade*				NS
1	20 (42)	8 (47)	12 (39)	
2	20 (42)	8 (47)	12 (39)	
3A	7 (15)	1 (6)	6 (19)	
Unknown	1 (1)	0 (0)	1 (3)	
*Ann Arbor Stage*				NS
III	24 (50)	8 (47)	16 (52)	
IV	24 (50)	9 (53)	15 (48)	
*B-Symptoms*				NS
No	31 (65)	11 (65)	20 (65)	
Yes	17 (35)	6 (35)	11 (35)	
*Bulky disease*				NS
No	30 (63)	10 (59)	20 (65)	
Yes	16 (33)	6 (35)	10 (32)	
Unknown	2 (4)	1 (6)	1 (3)	
*LDH-elevation*				NS
No	32 (67)	13 (76)	19 (61)	
Yes	16 (33)	4 (24)	12 (39)	
*FLIPI*				NS
Low/intermediate	20 (42)	9 (53)	11 (35)	
High	28 (58)	8 (47)	20 (65)	
*Anemia*				NS
No	43 (90)	15 (88)	28 (90)	
Yes	5 (10)	2 (12)	3 (10)	
*Nodal sites*				NS
≥4	5 (10)	0 (0)	5 (16)	
<4	43 (90)	17 (100)	26 (84)	
*POD24*				0.001
No	41 (85)	10 (59)	31 (100)	
Yes	7 (15)	7 (41)	0 (0)	
*Transformation*				NS
No	47 (98)	16 (94)	31 (100)	
Yes	1 (2)	1 (6)	0 (0)	
*Death*				NS
No	47 (98)	17 (100)	30 (97)	
Yes	1 (2)	0 (0)	1 (3)	

Abbreviations: FLIPI, follicular lymphoma international prognostic index; LDH, lactate dehydrogenase; NS, not significant; POD24, progression of disease within 24 months.

## Data Availability

Data included in the current study are available upon reasonable request to the corresponding authors.

## Supplementary Materials

The following supporting information can be downloaded at: https://www.mdpi.com/article/10.3390/cancers16193278/s1, Supplementary Methods; Table S1. Significantly differentially expressed proteins between sp-FL and np-FL samples; Table S2. Significantly differentially expressed proteins, LRG analysis; Table S3. Significantly differentially expressed proteins, HRG analysis; Table S4. Enrichment analysis; Table S5. Correlations of differentially expressed markers; Table S6. Staining parameters for immunohistochemical protocols; Figure S1. Exclusion of sample; Figure S2. Swimmer’s plot; Figure S3. PCA plot based on all identified proteins. Figure S4. Proteins identified from LRG and HRG analysis ability to separate the entire cohort; Figure S5. Representative images of STING1 and IDH2 staining. References [19,25,52,53,54,55,56,57,58,59,60,61] are cited in the Supplementary Materials.

## References

[1] Freedman A., Jacobsen E. (2020). Follicular lymphoma: 2020 update on diagnosis and management. Am. J. Hematol..

[2] Swerdlow S.H., Campo E., Pileri S.A., Harris N.L., Stein H., Siebert R., Advani R., Ghielmini M., Salles G.A., Zelenetz A.D. (2016). The 2016 revision of the World Health Organization classification of lymphoid neoplasms. Blood.

[3] Townsend W., Buske C., Cartron G., Cunningham D., Dyer M.J.S., Gribben J.G., Zhang Z., Rufibach K., Nielsen T., Herold M. (2020). Comparison of efficacy and safety with obinutuzumab plus chemotherapy versus rituximab plus chemotherapy in patients with previously untreated follicular lymphoma: Updated results from the phase III Gallium Study. J. Clin. Oncol..

[4] Liu Q., Silva A., Kridel R. (2021). Predicting early progression in follicular lymphoma. Ann. Lymphoma.

[5] Casulo C., Dixon J.G., Le-Rademacher J., Hoster E., Hochster H.S., Hiddemann W., Marcus R., Kimby E., Herold M., Sebban C. (2022). Validation of POD24 as a robust early clinical end point of poor survival in FL from 5225 patients on 13 clinical trials. Blood.

[6] Lipof J.J., Barr P.M. (2020). Early Progression of Follicular Lymphoma: Biology and Treatment. Hematol. Oncol. Clin..

[7] Russler-Germain D.A., Krysiak K., Ramirez C., Mosior M., Watkins M.P., Gomez F., Skidmore Z.L., Trani L., Gao F., Geyer S. (2023). Mutations associated with progression in follicular lymphoma predict inferior outcomes at diagnosis: Alliance A151303. Blood Adv..

[8] Sortais C., Lok A., Tessoulin B., Gastinne T., Mahé B., Dubruille V., Blin N., Touzeau C., Moreau A., Bossard C. (2020). Progression of disease within 2 years (POD24) is a clinically relevant endpoint to identify high-risk follicular lymphoma patients in real life. Ann. Hematol..

[9] Tobin J.W.D., Keane C., Gunawardana J., Mollee P., Birch S., Hoang T., Lee J., Li L., Huang L., Murigneux V. (2019). Progression of Disease within 24 Months in Follicular Lymphoma Is Associated With Reduced Intratumoral Immune Infiltration. J. Clin. Oncol..

[10] Wallace D., Casulo C. (2021). Early Progressing Follicular Lymphoma. Curr. Oncol. Rep..

[11] Jelicic J., Stauffer Larsen T., Bukumiric Z., Andjelic B. (2021). The clinical applicability of current prognostic models in follicular lymphoma: A systematic review. Crit. Rev. Oncol. Hematol..

[12] Bachy E., Maurer M.J., Habermann T.M., Gelas-Dore B., Maucort-Boulch D., Estell J.A., Van den Neste E., Bouabdallah R., Gyan E., Feldman A.L. (2018). A simplified scoring system in de novo follicular lymphoma treated initially with immunochemotherapy. Blood.

[13] Devan J., Janikova A., Mraz M. (2018). New concepts in follicular lymphoma biology: From BCL2 to epigenetic regulators and non-coding RNAs. Semin. Oncol..

[14] Federico M., Bellei M., Marcheselli L., Luminari S., Lopez-Guillermo A., Vitolo U., Pro B., Pileri S., Pulsoni A., Soubeyran P. (2009). Follicular lymphoma international prognostic index 2: A new prognostic index for follicular lymphoma developed by the international follicular lymphoma prognostic factor project. J. Clin. Oncol..

[15] Pastore A., Jurinovic V., Kridel R., Hoster E., Staiger A.M., Szczepanowski M., Pott C., Kopp N., Murakami M., Horn H. (2015). Integration of gene mutations in risk prognostication for patients receiving first-line immunochemotherapy for follicular lymphoma: A retrospective analysis of a prospective clinical trial and validation in a population-based registry. Lancet Oncol..

[16] Press O.W., Unger J.M., Rimsza L.M., Friedberg J.W., LeBlanc M., Czuczman M.S., Kaminski M., Braziel R.M., Spier C., Gopal A.K. (2013). A comparative analysis of prognostic factor models for follicular lymphoma based on a phase III trial of CHOP-rituximab versus CHOP + 131iodine–tositumomab. Clin. Cancer Res..

[17] Yang G., Mills M., Kim Y., Figura N.B., Doyle C., Oliver D., Grass G.D., Robinson T., Chavez J., Kim S. (2020). Enhancement of the Follicular Lymphoma International Prognostic Index (FLIPI) with lymphopenia (FLIPI-L): A predictor for overall survival and histologic transformation. Blood Cancer J..

[18] Mir F., Mattiello F., Grigg A., Herold M., Hiddemann W., Marcus R., Seymour J.F., Bolen C.R., Knapp A., Nielsen T. (2020). Follicular Lymphoma Evaluation Index (FLEX): A new clinical prognostic model that is superior to existing risk scores for predicting progression-free survival and early treatment failure after frontline immunochemotherapy. Am. J. Hematol..

[19] Enemark M.B.H., Wolter K., Campbell A.J., Andersen M.D., Sørensen E.F., Hybel T.E., Madsen C., Lauridsen K.L., Plesner T.L., Hamilton-Dutoit S.J. (2023). Proteomics identifies apoptotic markers as predictors of histological transformation in patients with follicular lymphoma. Blood Adv..

[20] Kamper P., Ludvigsen M., Bendix K., Hamilton-Dutoit S., Rabinovich G.A., Møller M.B., Nyengaard J.R., Honoré B., d’Amore F. (2011). Proteomic analysis identifies galectin-1 as a predictive biomarker for relapsed/refractory disease in classical Hodgkin lymphoma. Blood.

[21] Ludvigsen M., Campbell A.J., Enemark M.B., Hybel T.E., Karjalainen-Lindsberg M.-L., Beiske K., Bjerre M., Pedersen L.M., Holte H., Leppä S. (2023). Proteomics uncovers molecular features for relapse risk stratification in patients with diffuse large B-cell lymphoma. Blood Cancer J..

[22] Vase M., Ludvigsen M., Bendix K., Hamilton-Dutoit S., Mller M.B., Pedersen C., Pedersen G., Obel N., Larsen C.S., d’Amore F. (2016). Proteomic profiling of pretreatment serum from HIV-infected patients identifies candidate markers predictive of lymphoma development. Aids.

[23] Gonzalez M.W., Kann M.G. (2012). Chapter 4: Protein interactions and disease. PLoS Comput. Biol..

[24] Arboe B., El-Galaly T.C., Clausen M.R., Munksgaard P.S., Stoltenberg D., Nygaard M.K., Klausen T.W., Christensen J.H., Gørløv J.S., Brown Pde N. (2016). The Danish National Lymphoma Registry: Coverage and Data Quality. PLoS ONE.

[25] Szklarczyk D., Gable A.L., Nastou K.C., Lyon D., Kirsch R., Pyysalo S., Doncheva N.T., Legeay M., Fang T., Bork P. (2021). The STRING database in 2021: Customizable protein-protein networks, and functional characterization of user-uploaded gene/measurement sets. Nucleic Acids Res..

[26] Dave S.S., Wright G., Tan B., Rosenwald A., Gascoyne R.D., Chan W.C., Fisher R.I., Braziel R.M., Rimsza L.M., Grogan T.M. (2004). Prediction of survival in follicular lymphoma based on molecular features of tumor-infiltrating immune cells. N. Engl. J. Med..

[27] Zhu Y., An X., Zhang X., Qiao Y., Zheng T., Li X. (2019). STING: A master regulator in the cancer-immunity cycle. Mol. Cancer.

[28] Müller L., Aigner P., Stoiber D. (2017). Type I Interferons and Natural Killer Cell Regulation in Cancer. Front. Immunol..

[29] Dahal L.N., Dou L., Hussain K., Liu R., Earley A., Cox K.L., Murinello S., Tracy I., Forconi F., Steele A.J. (2017). STING Activation Reverses Lymphoma-Mediated Resistance to Antibody Immunotherapy. Cancer Res..

[30] Landmann R., Müller B., Zimmerli W. (2000). CD14, new aspects of ligand and signal diversity. Microbes Infect..

[31] Zhang R., Kang R., Tang D. (2022). STING1 in Different Organelles: Location Dictates Function. Front. Immunol..

[32] Martínez-Reyes I., Chandel N.S. (2021). Cancer metabolism: Looking forward. Nat. Rev. Cancer.

[33] Krell D., Assoku M., Galloway M., Mulholland P., Tomlinson I., Bardella C. (2011). Screen for IDH1, IDH2, IDH3, D2HGDH and L2HGDH mutations in glioblastoma. PLoS ONE.

[34] Cardaci S., Ciriolo M.R. (2012). TCA Cycle Defects and Cancer: When Metabolism Tunes Redox State. Int. J. Cell Biol..

[35] Kotredes K.P., Razmpour R., Lutton E., Alfonso-Prieto M., Ramirez S.H., Gamero A.M. (2019). Characterization of cancer-associated IDH2 mutations that differ in tumorigenicity, chemosensitivity and 2-hydroxyglutarate production. Oncotarget.

[36] Lemonnier F., Cairns R.A., Inoue S., Li W.Y., Dupuy A., Broutin S., Martin N., Fataccioli V., Pelletier R., Wakeham A. (2016). The IDH2 R172K mutation associated with angioimmunoblastic T-cell lymphoma produces 2HG in T cells and impacts lymphoid development. Proc. Natl. Acad. Sci. USA.

[37] Anderson N.M., Simon M.C. (2020). The tumor microenvironment. Curr. Biol..

[38] Newman J.S., Francis I.R., Kaminski M.S., Wahl R.L. (1994). Imaging of lymphoma with PET with 2-[F-18]-fluoro-2-deoxy-D-glucose: Correlation with CT. Radiology.

[39] Dupuy A., Lemonnier F., Fataccioli V., Martin-Garcia N., Robe C., Pelletier R., Poullot E., Moktefi A., Mokhtari K., Rousselet M.C. (2018). Multiple Ways to Detect IDH2 Mutations in Angioimmunoblastic T-Cell Lymphoma from Immunohistochemistry to Next-Generation Sequencing. J. Mol. Diagn..

[40] Heavican T.B., Bouska A., Yu J., Lone W., Amador C., Gong Q., Zhang W., Li Y., Dave B.J., Nairismägi M.L. (2019). Genetic drivers of oncogenic pathways in molecular subgroups of peripheral T-cell lymphoma. Blood.

[41] Holst J.M., Enemark M.B., Pedersen M.B., Lauridsen K.L., Hybel T.E., Clausen M.R., Frederiksen H., Møller M.B., Nørgaard P., Plesner T.L. (2021). Proteomic Profiling Differentiates Lymphoma Patients with and without Concurrent Myeloproliferative Neoplasia. Cancers.

[42] Calvert A.E., Chalastanis A., Wu Y., Hurley L.A., Kouri F.M., Bi Y., Kachman M., May J.L., Bartom E., Hua Y. (2017). Cancer-Associated IDH1 Promotes Growth and Resistance to Targeted Therapies in the Absence of Mutation. Cell Rep..

[43] Wang J.B., Dong D.F., Wang M.D., Gao K. (2014). IDH1 overexpression induced chemotherapy resistance and IDH1 mutation enhanced chemotherapy sensitivity in Glioma cells in vitro and in vivo. Asian Pac. J. Cancer Prev..

[44] Li J., He Y., Tan Z., Lu J., Li L., Song X., Shi F., Xie L., You S., Luo X. (2018). Wild-type IDH2 promotes the Warburg effect and tumor growth through HIF1α in lung cancer. Theranostics.

[45] Nawrocki S.T., Carew J.S., Maclean K.H., Courage J.F., Huang P., Houghton J.A., Cleveland J.L., Giles F.J., McConkey D.J. (2008). Myc regulates aggresome formation, the induction of Noxa, and apoptosis in response to the combination of bortezomib and SAHA. Blood.

[46] Gulmann C., Espina V., Petricoin E., Longo D.L., Santi M., Knutsen T., Raffeld M., Jaffe E.S., Liotta L.A., Feldman A.L. (2005). Proteomic analysis of apoptotic pathways reveals prognostic factors in follicular lymphoma. Clin. Cancer Res..

[47] Weinkauf M., Christopeit M., Hiddemann W., Dreyling M. (2007). Proteome- and microarray-based expression analysis of lymphoma cell lines identifies a p53-centered cluster of differentially expressed proteins in mantle cell and follicular lymphoma. Electrophoresis.

[48] Duś-Szachniewicz K., Rymkiewicz G., Agrawal A.K., Kołodziej P., Wiśniewski J.R. (2021). Large-Scale Proteomic Analysis of Follicular Lymphoma Reveals Extensive Remodeling of Cell Adhesion Pathway and Identifies Hub Proteins Related to the Lymphomagenesis. Cancers.

[49] O’Shea D., O’Riain C., Taylor C., Waters R., Carlotti E., Macdougall F., Gribben J., Rosenwald A., Ott G., Rimsza L.M. (2008). The presence of TP53 mutation at diagnosis of follicular lymphoma identifies a high-risk group of patients with shortened time to disease progression and poorer overall survival. Blood.

[50] Kridel R., Sehn L.H., Gascoyne R.D. (2017). Can histologic transformation of follicular lymphoma be predicted and prevented?. Blood.

[51] Deng Y., Ma J., Zhao S., Yang M., Sun Y., Zhang Q. (2023). Expression of glucose transporter-1 in follicular lymphoma affected tumor-infiltrating immunocytes and was related to progression of disease within 24 months. Transl. Oncol..

[52] Honoré B., Costa C. (2020). Proteomic Protocols for Differential Protein Expression Analyses. Xenotransplantation: Methods and Protocols.

[53] Zougman A., Selby P.J., Banks R.E. (2014). Suspension trapping (STrap) sample preparation method for bottom-up proteomics analysis. Proteomics.

[54] Cehofski L.J., Kojima K., Terao N., Kitazawa K., Thineshkumar S., Grauslund J., Vorum H., Honoré B. (2020). Aqueous Fibronectin Correlates with Severity of Macular Edema and Visual Acuity in Patients With Branch Retinal Vein Occlusion: A Proteome Study. Investig. Ophthalmol. Vis. Sci..

[55] Tyanova S., Temu T., Cox J. (2016). The MaxQuant computational platform for mass spectrometry-based shotgun proteomics. Nat. Protoc..

[56] Tyanova S., Temu T., Sinitcyn P., Carlson A., Hein M.Y., Geiger T., Mann M., Cox J. (2016). The Perseus computational platform for comprehensive analysis of (prote)omics data. Nat. Methods.

[57] Doncheva N.T., Morris J.H., Gorodkin J., Jensen L.J. (2019). Cytoscape StringApp: Network Analysis and Visualization of Proteomics Data. J. Proteome Res..

[58] Szklarczyk D., Gable A.L., Lyon D., Junge A., Wyder S., Huerta-Cepas J., Simonovic M., Doncheva N.T., Morris J.H., Bork P. (2019). STRING v11: Protein-protein association networks with increased coverage, supporting functional discovery in genome-wide experimental datasets. Nucleic Acids Res..

[59] Beck Enemark M., Monrad I., Madsen C., Lystlund Lauridsen K., Honoré B., Plesner T.L., Hamilton-Dutoit S.J., d’Amore F., Ludvigsen M. (2021). PD-1 Expression in Pre-Treatment Follicular Lymphoma Predicts the Risk of Subsequent High-Grade Transformation. OncoTargets Ther..

[60] Enemark M.B., Hybel T.E., Madsen C., Lauridsen K.L., Honoré B., Plesner T.L., Hamilton-Dutoit S., d’Amore F., Ludvigsen M. (2022). Tumor-Tissue Expression of the Hyaluronic Acid Receptor RHAMM Predicts Histological Transformation in Follicular Lymphoma Patients. Cancers.

[61] Hybel T.E., Vase M.Ø., Maksten E.F., Enemark M.B., Lauridsen K.L., Hamilton-Dutoit S., Andersen C., Møller M.B., Sørensen S.S., Jespersen B. (2021). Intratumoral expression of CD38 in patients with post-transplant lymphoproliferative disorder. Acta Oncol..

