# Supplementary material for: Proteomic Profiling Identifies Predictive Signatures for Progression Risk in Patients with Advanced-Stage Follicular Lymphoma
Source: Cancers (Basel). 2024 Sep 26;16(19):3278. doi: 10.3390/cancers16193278 (PMC11476298; doi:10.3390/cancers16193278)
Supplement: Supplementary file 1 [file cancers-16-03278-s001.zip › cancers-3215469-supplementary.pdf]

Supplementary Materials: Proteomic Profiling Identifies Predictive Signatures for Progression Risk in Patients with Advanced-Stage Follicular Lymphoma

Jonas Klejs Hemmingsen, Marie Hairing Enemark, Emma Frasez Sørensen, Kristina Lystlund Lauridsen, Stephen Jacques Hamilton-Dutoit, Robert Kridel, Bent Honoré and Maja Ludvigsen

Supplementary Methods

Sample Preparation for Mass Spectrometry

A total of 49 diagnostic tumor formalin-fixed, paraffin-embedded (FFPE) samples from patients diagnosed with advanced-stage FL were analyzed (*i.e.,* 18 sp-FL and 31 np-FL) using proteomic analysis by tandem mass tag (TMT) based mass spectrometry. Seven 10 μm sections of FFPE tissue from each sample were prepared for protein analysis essentially as previously described[1]. First, paraffin was removed with xylene followed by rehydration with decreasing levels of ethanol/water, then dried and dissolved in lysis buffer (5% SDS, 50 mM triethylammonium bicarbonate (TEAB), pH 8.5). The protein concentration was estimated using infrared spectrometry (Direct Detect Spec-trometer, Merck KGaA, Darmstadt, Germany)[1].

Sample preparation was performed with the suspension-trapping method[2] using the S-Trap™ micro columns from Protifi (Farmingdale, NY, USA), as previously described[3]. Samples were processed for labeling with TMT 10plex™ isobaric mass tagging kit (Thermo Fisher Scientific, Waltham, MA, USA) in 10 groups by using the 49 analytical samples together with 31 other sample all in all 80 samples. A standard sample was prepared by taking 3 μg peptide from each of 49 samples. This standard was labeled with reagent 126 and 131 in each of the groups leaving 8 reagents, 127N, 127C, 128N, 128C, 129N, 129C, 130N, 130C to label 8 samples in each of the 10 groups. Nine and a half micrograms from each sample and standard were used for TMT labeling with TMT 10Plex™ isobaric mass tagging kit (Thermo Fisher Scientific). Equal amounts of the 10 samples of TMT labeled peptides in each group were mixed in each of 10 tubes and dried. The mixtures were then resuspended in 300 μL of 0.1% trifluoroacetic acid and fractionated in 8 samples using a Pierce High pH Reversed-Phase Peptide Fractionation Kit (Thermo Fisher Scientific) performed as described by the manufacturer.

Quantification with Tandem Mass Tag-Based Mass Spectrometry

Half a microgram of each fraction was injected into a Dionex Ultimate 3000 RSCL nano LC system connected to an Orbitrap Fusion Tribrid mass spectrometer (Thermo Fisher Scientific Instruments). Liquid chromatography and MS was performed using the TMT synchronous precursor selection MS^3 method, with settings as follows. The labeled samples were loaded onto the trapping column (5 mm x 300 μm, C18 PepMap100, 5 μm, 100 Å, Thermo Fisher Scientific) with the flow setting of 30 μL per min. The nanoflow was 300 nL per min for the separation of peptides on the analytical column (500 nm x 75 μm PepMap RSLC, C18, 2 μm, 100 Å, Thermo Fisher Scientific). The applied buffers were buffer A (99.9% water and 0.1% formic acid) and buffer B (99.9%

acetonitrile and 0.1% formic acid). The gradient was performed over 213 min using a gradient of buffer B ranging from 2% to 80%.

The mass spectrometer was operated in the TMT synchronous precursors selection MS3 mode with full Orbitrap scans in the mass range of 350–1500 m/z obtained at a resolution of 120,000 with an AGC target of 2×10^5 and a maximum injection time of 50 ms. A dynamic exclusion of 60 s was applied. The mass spectrometer was set to trigger MS^2 acquisitions in each cycle using the linear ion trap with a collision-induced dissociation collision energy at 35% and an AGC target of 2×10^4 with a maximal injection time of 70 ms. Precursor ions, in the mass range of 400–1200 m/z, were isolated in the quadrupole set with an isolation window of 2 m/z. Up to five reporter ions were detected in MS^3 with synchronous precursor selection performed in the Orbitrap in the mass range of 100–500 m/z with the higher-energy collisional dissociation collision energy set to 65%, obtained at a resolution of 50,000 and an AGC target of 3×10^4 and a maximum injection time of 110 ms. Up to three replicas of each fraction were analyzed.

The raw files were entered and further analyzed in MaxQuant (MaxQuant version 1.6.3.4, Max Planck Institute of Biochemistry, Martinsried, Germany: https://maxquant.net/maxquant/)[4] using the UniProt *Homo sapiens* filtered and reviewed database (www.uniprot.org), downloaded March 9th 2021. The generated protein-groups file was entered into Perseus version 1.6.14.0 (Max Planck Institute of Biochemistry, Martinsried, Germany: https://maxquant.net/perseus/)[5]. The proteins were identified based on at least 2 unique peptides. The reporter intensities were $\log_2$ transformed. In each group the two channels with identical measured standards were averaged to one standard. In each group, the replica with the highest number of identifications was used for further analysis giving 10 groups with a total of 3,943 proteins identified across all samples. The 5 groups were filtered to contain only valid values in all of the standard determinations, *i.e.,* 1,940 proteins. This set was exported to excel for normalization of each reporter value in each of the groups relative to one of the groups *i.e.,* group 1. The normalized set of $\log_2$ transformed reporter values for the 49 samples, without the standards, were then used for further analyses. Importantly, one sample was excluded due to quality issues associated with the sample, which could have affected the analysis, as seen in the PCA plot (Supplementary Figure S1A). Moreover, the sample had 66 missing values compared to the mean number of missing values of 7.29, indicating a quality issue (Supplementary Figure S1B).

Bioinformatic analysis

Bioinformatic analysis was performed using the STRING database (string-db.org). Each protein's corresponding UniProt ID were submitted to the software. The first listed UniProt ID was used for the analyses, when more than one UniProt ID were identified.

All 99 significantly differentially expressed proteins ($P < 0.05$) identified were entered into the STRING software tool and analyzed. The database was assessed using the String App (version 1.7.0) in Cytoscape (version 3.9.1)[6-8]. The minimum required interaction was set to confidence = 0.2, including both functional and physical interactions. On the entire network of proteins, enrichment analysis was performed. Here, only terms from Gene Ontology (GO) Biological processes, GO molecular functions, Kyoto Encyclopedia of Genes and Genomes pathways (KEGG), Reactome pathways, and WikiPathways. The false

discovery rate was set to 5%. The option to remove redundant terms was enabled with the redundancy cut-off set to 0.5.

Immunohistochemical staining of selected proteins

From the MS-based proteomics, two proteins were selected for evaluation using IHC, stimulator of interferon genes 1 (STING1) and isocitrate hydrogenase 2 (IDH2). Immunohistochemical staining was performed on 4 μm FFPE sections from whole tissue using the Ventana Benchmark Ultra automated staining system (Ventana Medical Systems, Roche, Oro Valley, AZ, USA). Slides were deparaffinized with EZ Prep solution (Ventana, 950-102), followed by blocking of endogenous peroxidase activity using the OptiView DAB IHC Detection Kit (Ventana, 760-700)[9-12]. Heat induced epitope retrieval (HIER) was performed by heating slides to 100°C in ULTRA Cell Conditioning Solution 1 (CC1, Ventana, 950-224). Next, primary antibody was diluted to optimal dilution in REAL™ antibody diluent (Dako, S202230-2) and added to the tissue and incubated at 37°C. Visualization was done using the OptiView IHC DAB Detection Kit (Ventana, 760-700) with nuclear counterstaining by hematoxylin. Sections of appendix, tonsil liver, and pancreas were included on all slides as controls. Staining protocols are summarized in Supplementary Table S6.

Digital image analysis

All stained slides were scanned on the NanoZoomer 2.0HT (Hamamatsu, Shizouka, Japan) at a magnification of 20x. The scanned images were analyzed using the Visiopharm 2020.08 system (Visiopharm A/S Hoersholm, Denmark). Here, areas for staining quantification were defined by manual outlining of regions of interest (ROI) on each digitized whole tissue section. In the ROIs, areas of non-lymphoid tissue and technical artefacts were excluded. Analysis protocol packages were then designed to quantify the expression levels of each marker, as previously described[11].

Quantification results from staining were expressed as area fractions (AFs), calculated as the ratio of the stained area to the overall area within the ROI.

References

1. Honoré B. Proteomic Protocols for Differential Protein Expression Analyses. In: Costa C, ed. *Xenotransplantation: Methods and Protocols*. Springer US; 2020:47-58.
2. Zougman A, Selby PJ, Banks RE. Suspension trapping (STrap) sample preparation method for bottom-up proteomics analysis. *Proteomics*. May 2014;14(9):1006-0. doi:10.1002/pmic.201300553
3. Cehofski LJ, Kojima K, Terao N, et al. Aqueous Fibronectin Correlates With Severity of Macular Edema and Visual Acuity in Patients With Branch Retinal Vein Occlusion: A Proteome Study. *Invest Ophthalmol Vis Sci*. Dec 1 2020;61(14):6. doi:10.1167/iovs.61.14.6
4. Tyanova S, Temu T, Cox J. The MaxQuant computational platform for mass spectrometry-based shotgun proteomics. *Nat Protoc*. Dec 2016;11(12):2301-2319. doi:10.1038/nprot.2016.136
5. Tyanova S, Temu T, Sinitcyn P, et al. The Perseus computational platform for comprehensive analysis of (prote)omics data. *Nature Methods*. 2016/09/01 2016;13(9):731-740. doi:10.1038/nmeth.3901
6. Doncheva NT, Morris JH, Gorodkin J, Jensen LJ. Cytoscape StringApp: Network Analysis and Visualization of Proteomics Data. *J Proteome Res*. Feb 1 2019;18(2):623-632. doi:10.1021/acs.jproteome.8b00702
7. Szklarczyk D, Gable AL, Lyon D, et al. STRING v11: protein-protein association networks with increased coverage, supporting functional discovery in genome-wide experimental datasets. *Nucleic Acids Res*. Jan 8 2019;47(D1):D607-d613. doi:10.1093/nar/gky1131
8. Szklarczyk D, Gable AL, Nastou KC, et al. The STRING database in 2021: customizable protein-protein networks, and functional characterization of user-uploaded gene/measurement sets. *Nucleic Acids Res*. Jan 8 2021;49(D1):D605-d612. doi:10.1093/nar/gkaa1074
9. Beck Enemark M, Monrad I, Madsen C, et al. PD-1 Expression in Pre-Treatment Follicular Lymphoma Predicts the Risk of Subsequent High-Grade Transformation. *Onco Targets Ther*. 2021;14:481-489. doi:10.2147/ott.S289337

10. Enemark MB, Hybel TE, Madsen C, et al. Tumor-Tissue Expression of the Hyaluronic Acid Receptor RHAMM Predicts Histological Transformation in Follicular Lymphoma Patients. *Cancers (Basel)*. Mar 4 2022;14(5)doi:10.3390/cancers14051316
11. Enemark MBH, Wolter K, Campbell AJ, et al. Proteomics identifies apoptotic markers as predictors of histological transformation in patients with follicular lymphoma. *Blood Advances*. 2023;7(24):7418-7432. doi:10.1182/bloodadvances.2023011299
12. Hybel TE, Vase M, Maksten EF, et al. Intratumoral expression of CD38 in patients with post-transplant lymphoproliferative disorder. *Acta Oncol*. Dec 2021;60(12):1637-1642. doi:10.1080/0284186x.2021.1973093

Supplementary Tables

Supplementary Table S1. Significantly differentially expressed proteins between sp-FL and np-FL samples

Fold changes (sp-FL/np-FL)	P-value	Gene name	Protein name
**Upregulated**			
0.24	0.031	*BIRC6*	Baculoviral IAP repeat-containing protein 6
0.17	0.036	*C16orf62*	UPF0505 protein C16orf62
**Downregulated**			
-0.17	0.037	*TSC22D4*	TSC22 domain family protein 4
-0.23	0.047	*UBL4A*	Ubiquitin-like protein 4A
-0.23	0.028	*NASP*	Nuclear autoantigenic sperm protein
-0.23	0.049	*ANXA11*	Annexin A11
-0.25	0.045	*CAPZA2*	F-actin-capping protein subunit alpha-2
-0.25	0.035	*GCLC*	Glutamate--cysteine ligase catalytic subunit
-0.26	0.044	*ACTL6A*	Actin-like protein 6A
-0.27	0.037	*HN1L*	Hematological and neurological expressed 1-like protein
-0.27	0.048	*ELMO1*	Engulfment and cell motility protein 1
-0.27	0.015	*AP2B1*	AP-2 complex subunit beta
-0.28	0.033	*HSPA1B*	Heat shock 70 kDa protein 1B
-0.29	0.024	*PSMF1*	Proteasome inhibitor PI31 subunit
-0.30	0.019	*MTPN*	Myotrophin
-0.30	0.043	*TBC1D10C*	Carabin
-0.30	0.016	*RPA3*	Replication protein A 14 kDa subunit
-0.31	0.038	*ARF5*	ADP-ribosylation factor 5
-0.31	0.048	*ERO1L*	ERO1-like protein alpha
-0.31	0.040	*FH*	Fumarate hydratase, mitochondrial
-0.31	0.022	*YWHAE*	14-3-3 protein epsilon
-0.31	0.041	*ARHGAP30*	Rho GTPase-activating protein 30
-0.32	0.045	*PPIB*	Peptidyl-prolyl cis-trans isomerase B
-0.32	0.023	*BSG*	Basigin
-0.32	0.025	*YWHAG*	14-3-3 protein gamma
-0.32	0.047	*PNP*	Purine nucleoside phosphorylase
-0.32	0.048	*ITPA*	Inosine triphosphate pyrophosphatase
-0.33	0.042	*CHMP1A*	Charged multivesicular body protein 1a
-0.33	0.048	*HEXA*	Beta-hexosaminidase subunit alpha
-0.33	0.042	*HYOU1*	Hypoxia up-regulated protein 1
-0.34	0.040	*CD2BP2*	CD2 antigen cytoplasmic tail-binding protein 2
-0.34	0.034	*MLEC*	Malectin
-0.35	0.024	*AP1S1*	AP-1 complex subunit sigma-1A
-0.35	0.042	*VAPB*	Vesicle-associated membrane protein-associated protein B/C
-0.37	0.025	*CANX*	Calnexin

-0.37	0.019	*RPRD1B*	Regulation of nuclear pre-mRNA domain-containing protein 1B
-0.37	0.037	*NDUFV1*	NADH dehydrogenase flavoprotein 1, mitochondrial
-0.38	0.025	*STX5*	Syntaxin-5
-0.38	0.021	*RAB27A*	Ras-related protein Rab-27A
-0.38	0.038	*CASP4*	Caspase-4
-0.38	0.017	*GOLPH3*	Golgi phosphoprotein 3
-0.39	0.014	*SRRM2*	Serine/arginine repetitive matrix protein 2
-0.39	0.040	*SSR1*	Translocon-associated protein subunit alpha
-0.39	0.049	*LRRC59*	Leucine-rich repeat-containing protein 59
-0.40	0.012	*PPIA*	Peptidyl-prolyl cis-trans isomerase A
-0.40	0.034	*SAR1A*	GTP-binding protein SAR1a
-0.40	0.019	*PGRMC2*	Membrane-associated progesterone receptor component 2
-0.40	0.046	*SCARB2*	Lysosome membrane protein 2
-0.40	0.023	*HSPA5*	78 kDa glucose-regulated protein
-0.41	0.038	*COX4I1*	Cytochrome c oxidase subunit 4 isoform 1, mitochondrial
-0.41	0.011	*GLUL*	Glutamine synthetase
-0.41	0.029	*SLC25A1*	Tricarboxylate transport protein. mitochondrial
-0.41	0.046	*METTL7A*	Methyltransferase-like protein 7A
-0.41	0.032	*CIRBP*	Cold-inducible RNA-binding protein
-0.42	0.038	*CALD1*	Caldesmon
-0.42	0.041	*SCP2*	Non-specific lipid-transfer protein
-0.43	0.031	*IDH3A*	Isocitrate dehydrogenase [NAD] subunit alpha, mitochondrial
-0.43	0.043	*CFB*	Complement factor B
-0.43	0.014	*SERPINB6*	Serpin B6
-0.44	0.004	*SRI*	Sorcin
-0.45	0.046	*THRAP3*	Thyroid hormone receptor-associated protein 3
-0.46	0.020	*DLD*	Dihydrolipoyl dehydrogenase, mitochondrial
-0.46	0.022	*GBP2*	Interferon-induced guanylate-binding protein 2
-0.46	0.035	*ANXA1*	Annexin A1
-0.47	0.039	*SDHA*	Succinate dehydrogenase flavoprotein subunit, mitochondrial
-0.47	0.035	*MDH2*	Malate dehydrogenase, mitochondrial
-0.47	0.044	*CTSD*	Cathepsin D;Cathepsin D light chain;Cathepsin D heavy chain
-0.48	0.029	*RCN1*	Reticulocalbin-1
-0.48	0.021	*SEC61A1*	Protein transport protein Sec61 subunit alpha isoform 1
-0.48	0.048	*DUT*	Deoxyuridine 5-triphosphate nucleotidohydrolase, mitochondrial
-0.48	0.010	*SRPRB*	Signal recognition particle receptor subunit beta
-0.49	0.022	*DBR1*	Lariat debranching enzyme
-0.50	0.048	*APEX1*	DNA-(apurinic or apyrimidinic site) lyase, mitochondrial
-0.50	0.046	*GLUD1*	Glutamate dehydrogenase 1, mitochondrial
-0.50	0.015	*PDIA4*	Protein disulfide-isomerase A4
-0.52	0.037	*DSP*	Desmoplakin

-0.52	0.045	*HMGB2*	High mobility group protein B2
-0.53	0.015	*CD14*	Monocyte differentiation antigen CD14
-0.53	0.032	*VTN*	Vitronectin
-0.54	0.015	*STING1*	Stimulator of interferon genes protein
-0.56	0.017	*TGM2*	Protein-glutamine gamma-glutamyltransferase 2
-0.56	0.006	*GSTK1*	Glutathione S-transferase kappa 1
-0.60	0.006	*PMVK*	Phosphomevalonate kinase
-0.60	0.014	*GIMAP1*	GTPase IMAP family member 1
-0.62	0.043	*UCHL1*	Ubiquitin carboxyl-terminal hydrolase isozyme L1
-0.63	0.033	*FN1*	Fibronectin
-0.65	0.017	*FGB*	Fibrinogen beta chain
-0.65	0.012	*S100A11*	Protein S100-A11;Protein S100-A11, N-terminally processed
-0.66	0.013	*IGLL5*	Immunoglobulin lambda-like polypeptide 5
-0.68	0.003	*IDH2*	Isocitrate dehydrogenase [NADP], mitochondrial
-0.69	0.024	*CR2*	Complement receptor type 2
-0.73	0.018	*LGALS7*	Galectin-7
-0.79	0.026	*S100A8*	Protein S100-A8
-0.79	0.012	*FGA*	Fibrinogen alpha chain
-0.80	0.036	*S100A9*	Protein S100-A9
-0.83	0.028	*CALML3*	Calmodulin-like protein 3
-0.85	0.012	*SERPINB3*	Serpin B3
-0.96	0.002	*FGG*	Fibrinogen gamma chain
-1.28	0.001	*CALML5*	Calmodulin-like protein 5

Supplementary Table S2. Significantly differentially expressed proteins, LRG analysis

**Fold changes (sp-FL/np-FL)**	**P-value**	**Gene name**	**Protein name**
**Upregulated**			
0.24	0.031	*BIRC6*	Baculoviral IAP repeat-containing protein 6
0.30	0.031	*TBCB*	Tubulin-folding cofactor B
0.31	0.017	*C16orf62*	UPF0505 protein C16orf62
0.34	0.044	*TIAL1*	Nucleolysin TIAR
0.36	0.047	*APEH*	Acylamino-acid-releasing enzyme
0.36	0.033	*RPS3*	40S ribosomal protein S3
0.37	0.011	*AHSA1*	Activator of 90 kDa heat shock protein ATPase homolog 1
0.37	0.025	*HDAC1*	Histone deacetylase 1
0.38	0.010	*FAM49B*	Protein FAM49B
0.39	0.021	*RTN4*	Reticulon-4
0.39	0.043	*STK38*	Serine/threonine-protein kinase 38
0.39	0.029	*RAB2A*	Ras-related protein Rab-2A
0.40	0.024	*SFXN3*	Sideroflexin-3

0.40	0.013	*RAB5A*	Ras-related protein Rab-5A
0.40	0.007	*DDOST*	Dolichyl-diphosphooligosaccharide--protein glycosyl transferase 48 kDa subunit
0.40	0.046	*ITPR1*	Inositol 1.4.5-trisphosphate receptor type 1
0.41	0.036	*RPL18A*	60S ribosomal protein L18a
0.41	0.016	*RAB7A*	Ras-related protein Rab-7a
0.42	0.033	*EIF4E*	Eukaryotic translation initiation factor 4E
0.42	0.034	*ACAA1*	3-ketoacyl-CoA thiolase. peroxisomal
0.43	0.016	*LYPLA1*	Acyl-protein thioesterase 1
0.43	0.008	*PLEK*	Pleckstrin
0.44	0.040	*ELAVL1*	ELAV-like protein 1
0.44	0.022	*EHD4*	EH domain-containing protein 4
0.44	0.001	*PDDC1*	Parkinson disease 7 domain-containing protein 1
0.45	0.048	*NDUFA2*	NADH dehydrogenase [ubiquinone] 1 alpha subcom plex subunit 2
0.45	0.018	*RAB1A*	Ras-related protein Rab-1A
0.46	0.040	*CLPP*	ATP-dependent Clp protease proteolytic subunit, Mito chondrial
0.46	0.047	*CD97*	CD97 antigen
0.47	0.050	*NPM1*	Nucleophosmin
0.49	0.006	*RPL35*	60S ribosomal protein L35
0.49	0.008	*RAB9A*	Ras-related protein Rab-9A
0.49	0.033	*SRSF5*	Serine/arginine-rich splicing factor 5
0.50	0.012	*RPL21*	60S ribosomal protein L21
0.51	0.003	*RAB21*	Ras-related protein Rab-21
0.53	0.008	*DAD1*	Dolichyl-diphosphooligosaccharide--protein glycosyl transferase subunit DAD1
0.54	0.048	*SCCPDH*	Saccharopine dehydrogenase-like oxidoreductase
0.55	0.029	*PDIA6*	Protein disulfide-isomerase A6
0.55	0.008	*RAB4B*	Ras-related protein Rab-4B
0.56	0.020	*FUCA1*	Tissue alpha-L-fucosidase
0.56	0.028	*VPS33B*	Vacuolar protein sorting-associated protein 33B
0.57	0.018	*CD47*	Leukocyte surface antigen CD47
0.58	0.003	*MAP2K1*	Dual specificity mitogen-activated protein kinase kinase 1
0.61	0.016	*CHID1*	Chitinase domain-containing protein 1
0.63	0.018	*ERGIC1*	Endoplasmic reticulum-Golgi intermediate compart ment protein 1
0.66	0.006	*CYB5R3*	NADH-cytochrome b5 reductase 3
0.70	0.024	*SYNGR2*	Synaptogyrin-2
0.77	0.017	*IL4I1*	L-amino-acid oxidase
0.77	0.001	*LIMA1*	LIM domain and actin-binding protein 1
0.85	0.023	*MX1*	Interferon-induced GTP-binding protein Mx1
0.93	0.012	*CD27*	CD27 antigen

Downregulated

-0.39	0.031	*GLO1*	Lactoylglutathione lyase
-0.54	0.008	*PPP3CA*	Serine/threonine-protein phosphatase 2B catalytic sub unit alpha isoform
-0.63	0.021	*CCBL2*	Kynurenine--oxoglutarate transaminase 3
-1.23	0.011	*CR2*	Complement receptor type 2
-1.23	0.014	*UCHL1*	Ubiquitin carboxyl-terminal hydrolase isozyme L1

Supplementary Table S3. Significantly differentially expressed proteins, HRG analysis

Fold changes (sp-FL/np-FL)	P-value	Gene name	Protein name
**Upregulated**			
0.23	0.042	*LCP1*	Plastin-2
0.26	0.049	*PSMB2*	Proteasome subunit beta type-2
0.26	0.047	*TAP2*	Antigen peptide transporter 2
0.26	0.048	*OXSR1*	Serine/threonine-protein kinase OSR1
0.27	0.041	*WDR44*	WD repeat-containing protein 44
0.28	0.046	*VBP1*	Prefoldin subunit 3
0.29	0.023	*PSME1*	Proteasome activator complex subunit 1
0.29	0.028	*TBC1D9B*	TBC1 domain family member 9B
0.30	0.026	*VPS13C*	Vacuolar protein sorting-associated protein 13C
0.32	0.047	*HUWE1*	E3 ubiquitin-protein ligase HUWE1
0.32	0.039	*SCLY*	Selenocysteine lyase
0.33	0.045	*PSMB3*	Proteasome subunit beta type-3
0.34	0.040	*ACTR3*	Actin-related protein 3
0.34	0.022	*PGK1*	Phosphoglycerate kinase 1
0.34	0.030	*SEC23B*	Protein transport protein Sec23B
0.34	0.017	*IL16*	Interleukin-16
0.34	0.013	*NADSYN1*	Glutamine-dependent NAD(+) synthetase
0.34	0.037	*TWF2*	Twinfilin-2
0.37	0.032	*CP*	Ceruloplasmin
0.39	0.023	*STK26*	Serine/threonine-protein kinase 26
0.39	0.042	*RP2*	Protein XRP2
0.40	0.041	*FUK*	L-fucose kinase
0.41	0.019	*MMP9*	Matrix metalloproteinase-9;67 kDa matrix metallopro teinase-9;82 kDa matrix metalloproteinase-9
0.41	0.050	*GLO1*	Lactoylglutathione lyase
0.43	0.009	*BIRC6*	Baculoviral IAP repeat-containing protein 6
0.44	0.035	*PPP3CB*	Serine/threonine-protein phosphatase 2B catalytic subu nit beta isoform
0.44	0.042	*PTPRC*	Receptor-type tyrosine-protein phosphatase C
0.45	0.003	*SIPA1*	Signal-induced proliferation-associated protein 1
0.45	0.023	*ESD*	S-formylglutathione hydrolase
0.45	0.048	*NUDT2*	Bis(5-nucleosyl)-tetraphosphatase [asymmetrical]
0.46	0.039	*PIN4*	Peptidyl-prolyl cis-trans isomerase NIMA-interacting 4
0.46	0.010	*CORO1A*	Coronin-1A
0.46	0.013	*LTA4H*	Leukotriene A-4 hydrolase
0.48	0.026	*LRRC40*	Leucine-rich repeat-containing protein 40
0.48	0.024	*C9orf64*	UPF0553 protein C9orf64
0.49	0.038	*ARPC1A*	Actin-related protein 2/3 complex subunit 1A

0.49	0.005	*CNP*	2,3-cyclic-nucleotide 3-phosphodiesterase
0.50	0.045	*BLMH*	Bleomycin hydrolase
0.55	0.009	*HERC4*	Probable E3 ubiquitin-protein ligase HERC4
0.56	0.040	*CAT*	Catalase
0.58	0.007	*POLDIP2*	Polymerase delta-interacting protein 2
0.58	0.034	*SEPT8*	Septin-8
0.64	0.040	*PPP3CA*	Serine/threonine-protein phosphatase 2B catalytic subu nit alpha isoform
0.68	0.042	*RALGPS2*	Ras-specific guanine nucleotide-releasing factor RalGPS2
0.93	0.001	*DSTN*	Destrin
1.04	0.015	*HBD*	Hemoglobin subunit delta
1.19	0.041	*TCL1A*	T-cell leukemia/lymphoma protein 1A
**Downregulated**			
-0.39	0.036	*RAB27A*	Ras-related protein Rab-27A
-0.47	0.013	*PDIA6*	Protein disulfide-isomerase A6
-0.47	0.031	*IDH2*	Isocitrate dehydrogenase [NADP], mitochondrial
-0.54	0.045	*PDIA4*	Protein disulfide-isomerase A4

Supplementary Table S4. Enrichment analysis

Category	Associated function	FDR value	P-value	#Proteins
Reactome Pathways	Innate Immune System	3.51E-10	1.54E-13	28
Reactome Pathways	MyD88 deficiency (TLR2/4)	6.78E-7	1.19E-9	6
WikiPathways	TCA cycle (aka Krebs or citric acid cycle)	1.23E-6	1.58E-9	6
WikiPathways	VEGFA-VEGFR2 signaling	1.0E-4	2.69E-7	13
Reactome Pathways	Disease	1.3E-4	7.14E-7	25
Reactome Pathways	GRB2:SOS provides linkage to MAPK signaling for Integrins	3.1E-4	2.07E-6	4
KEGG Pathways	Protein processing in endoplasmic reticulum	3.6E-4	2.14E-6	8
GO Biological Process	Transport	4.9E-4	7.86E-8	42
GO Biological Process	Movement in host	4.9E-4	5.11E-8	9
GO Biological Process	Biological process involved in interspecies interaction between organisms	4.9E-4	5.91E-8	25
GO Biological Process	Positive regulation of protein secretion	4.9E-4	3.15E-8	9
GO Biological Process	Dicarboxylic acid metabolic process	5.9E-4	5.62E-7	7
GO Biological Process	Negative regulation of extrinsic apoptotic signaling pathway	6.9E-4	8.42E-7	7
GO Biological Process	Response to stress	7.0E-4	8.98E-7	37
GO Molecular Function	Cell adhesion molecule binding	7.2E-4	1.46E-7	15
GO Molecular Function	Binding	9.2E-4	3.73E-7	86
GO Biological Process	Protein transport	9.6E-4	1.41E-6	20
WikiPathways	Complement system	0.0015	1.16E-5	6
GO Biological Process	Cell-cell adhesion	0.0019	3.49E-6	13
Reactome Pathways	Adaptive Immune System	0.0028	2.62E-5	14

GO Biological Process	Granulocyte chemotaxis	0.0029	6.38E-6	6
GO Biological Process	Maintenance of location in cell	0.0047	1.22E-5	6
GO Biological Process	Autocrine signaling	0.0051	1.38E-5	3
Reactome Pathways	SARS-CoV-1 Infection	0.0064	8.75E-5	6
Reactome Pathways	Programmed Cell Death	0.0065	9.14E-5	7
Reactome Pathways	IRE1alpha activates chaperones	0.0086	1.5E-4	4
GO Molecular Function	Small molecule binding	0.0098	8.99E-6	29
GO Biological Process	Chaperone-mediated protein folding	0.0105	3.75E-5	5
GO Biological Process	Protein secretion	0.0113	4.2E-5	6
GO Biological Process	Protein folding in endoplasmic reticulum	0.0113	4.12E-5	3
GO Biological Process	Cellular catabolic process	0.0113	4.33E-5	21
Reactome Pathways	Metabolism	0.0115	2.1E-4	23
GO Biological Process	Peptide cross-linking	0.0122	4.97E-5	4
GO Biological Process	Wound healing	0.0126	5.38E-5	9
KEGG Pathways	Parkinson disease	0.0139	2.1E-4	7
GO Biological Process	Cell redox homeostasis	0.0153	7.2E-5	4
GO Biological Process	Tricarboxylic acid metabolic process	0.0159	7.61E-5	3
GO Biological Process	Response to inorganic substance	0.0164	7.76E-5	11
GO Molecular Function	Chaperone binding	0.0179	2.19E-5	6
Reactome Pathways	Cellular responses to stress	0.0186	3.7E-4	12
WikiPathways	Urea cycle and associated pathways	0.0195	2.5E-4	3
GO Biological Process	Regulation of apoptotic process	0.0201	1.0E-4	19
Reactome Pathways	The role of Nef in HIV-1 replication and disease pathogenesis	0.0206	4.3E-4	3
Reactome Pathways	MHC class II antigen presentation	0.0206	4.2E-4	5
GO Molecular Function	Unfolded protein binding	0.0216	3.06E-5	6
Reactome Pathways	Regulation of HSF1-mediated heat shock response	0.0216	4.7E-4	4
GO Biological Process	Organonitrogen compound metabolic process	0.0221	1.2E-4	42
Reactome Pathways	Vesicle-mediated transport	0.0233	5.2E-4	11
GO Biological Process	Cellular response to chemical stimulus	0.0242	1.4E-4	27
GO Biological Process	Regulation of transport	0.0244	1.4E-4	21
GO Biological Process	Regulation of hydrolase activity	0.0248	1.5E-4	15
GO Biological Process	Defense response to bacterium	0.0262	1.7E-4	8
KEGG Pathways	Glyoxylate and dicarboxylate metabolism	0.0268	4.8E-4	3
GO Molecular Function	Calcium-dependent protein binding	0.0322	7.19E-5	5
GO Molecular Function	Organic acid binding	0.0346	8.42E-5	6
GO Biological Process	Protein targeting	0.0354	2.4E-4	7
GO Biological Process	Cell adhesion molecule production	0.0354	2.4E-4	2
GO Biological Process	Response to temperature stimulus	0.0368	2.6E-4	6
GO Biological Process	Phagocytosis	0.0376	2.7E-4	6
GO Biological Process	Regulation of biological quality	0.0381	2.7E-4	33
Reactome Pathways	Hemostasis	0.0398	9.6E-4	10
WikiPathways	Prolactin signaling pathway	0.0436	6.7E-4	4
GO Biological Process	Response to organic cyclic compound	0.0479	3.7E-4	13

Supplementary Table S5. Correlations of differentially expressed markers

	**STING1**	**IDH2**
**STING1**		
**IDH2**	(**P < 0.001**, ρ = 0.47)	
**Age**	(**P = 0.014**, ρ = 0.35)	NS
**≥4 Nodal-sites**	(**P = 0.033**, ρ = -0.31)	NS
**POD24**	(P = 0.093, ρ = -0.25)	(P = 0.065, ρ = -0.27)
**FL grade**	NS	(**P = 0.031**, ρ = 0.32)
**B-symptoms**	NS	(**P = 0.018**, ρ = -0.34)
**Progression**	NS	(**P = 0.032**, ρ = -0.31)

P-values in bold are significant. NS, not significant.

Supplementary Table S6. Staining parameters for immunohistochemical protocols

**Target protein**	**Antibody**	**Dilution**	**Heat-Induced Epitope Retrieval (min)**	**Primary Antibody Incubation (min)**	**Buffer for HIER**
**STING1**	Anti-STING1 (19851-1-AP, ProteinTech)	1:2000	32	32	CC1
**IDH2**	Anti-IDH2 (15932-1-AP, ProteinTech)	1:200	64	32	CC1

CC1 = cell conditioning solution. All antibodies were validated for IHC, polyclonal and produced in rabbit.

Supplementary figures

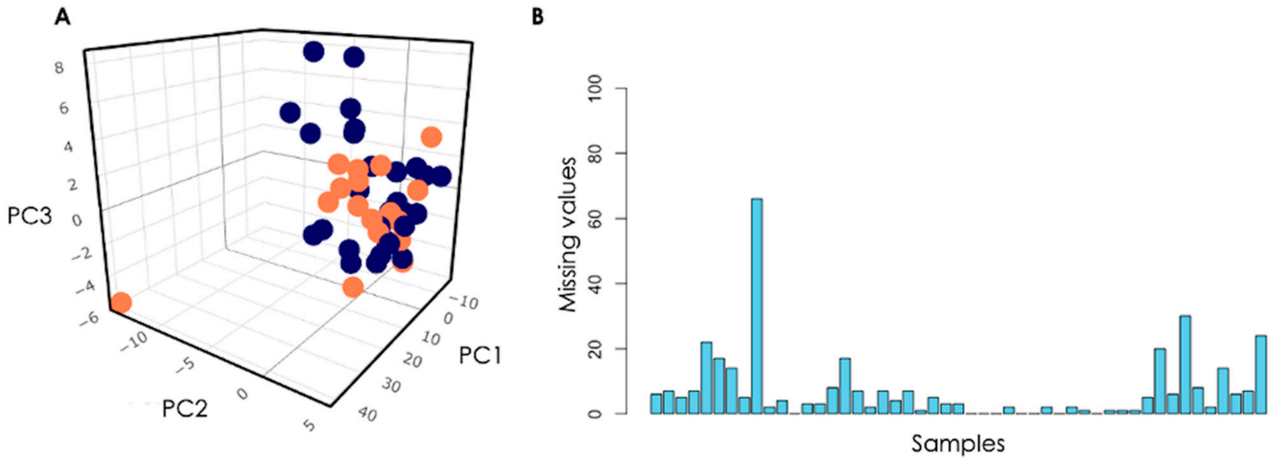

Supplementary Figure S1: Exclusion of sample. ***A)*** *PCA plot before excluding the sample. The outlier is seen in the bottom left corner.* ***B)*** *Barplot showing the number of missing values from each sample. The mean number of missing values is 7.29, whereas the outlier has 66 missing values.*

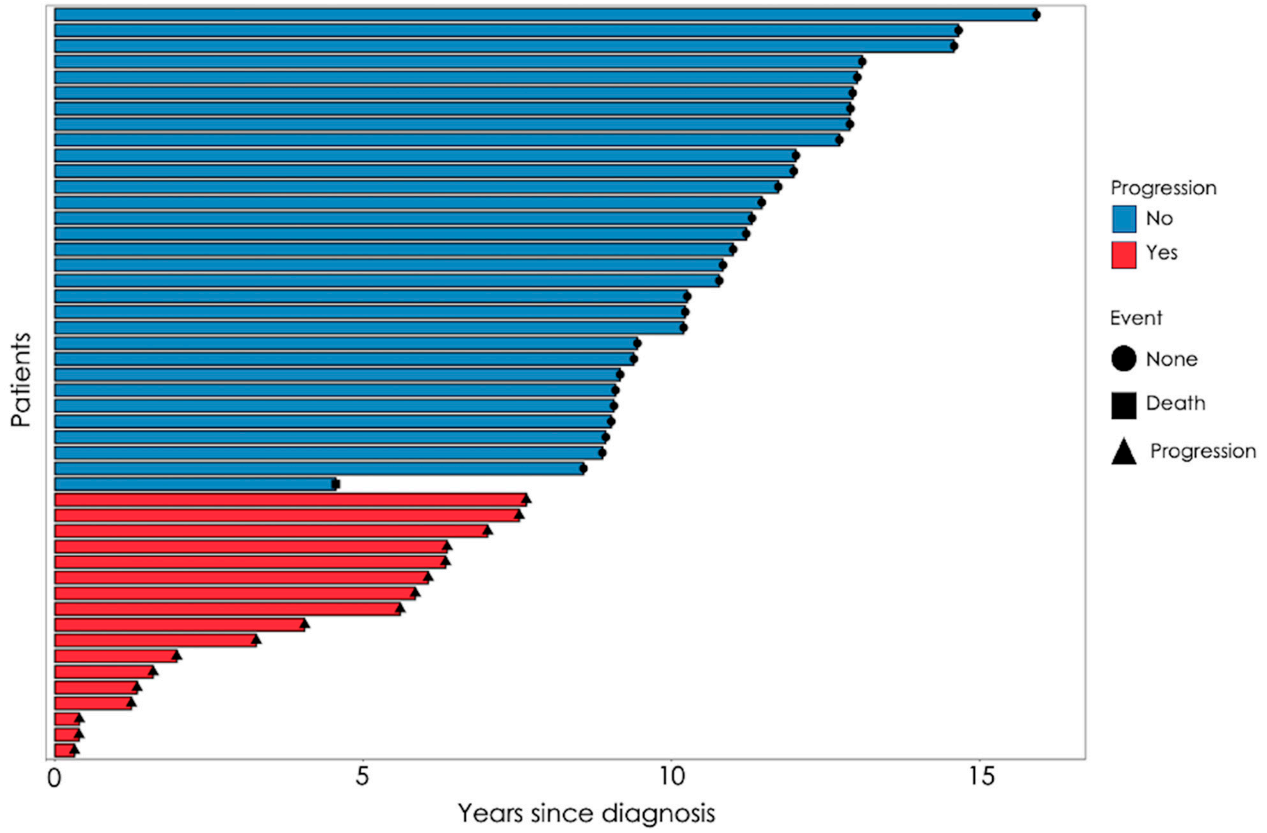

Supplementary Figure S2: Swimmer's plot. *Each bar in the plot represents a patient. Blue bars represent patients who did not experience progression during the study period. Red bars represents patients who experienced progression. One patient died after 4.5 years, potentially experiencing subsequent progression before the last date of follow-up.*

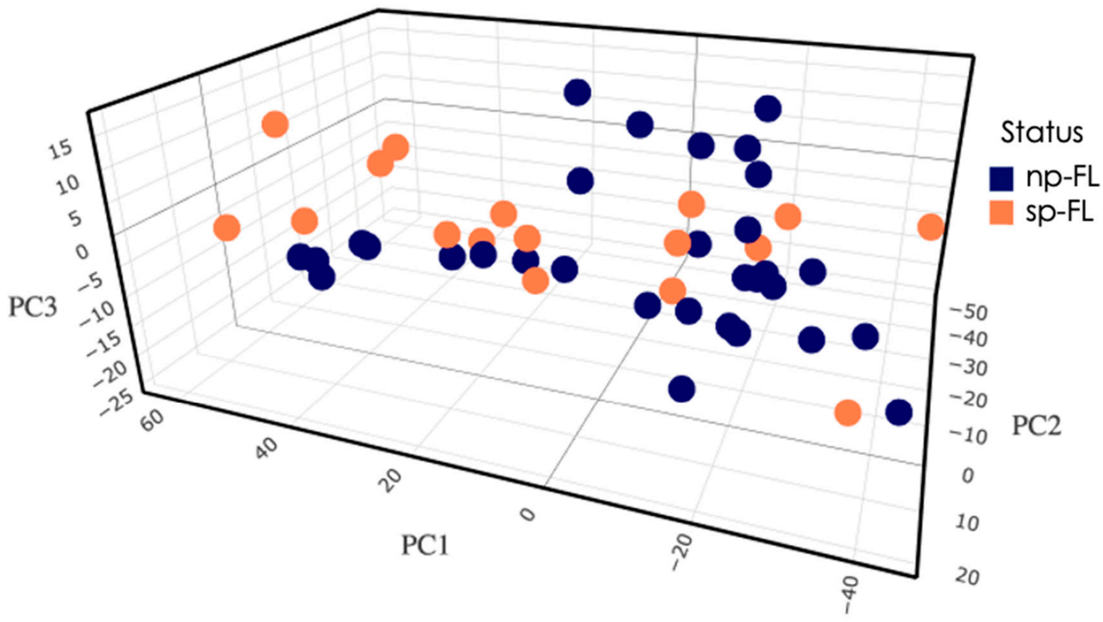

Supplementary Figure S3: PCA plot based on all identified proteins.

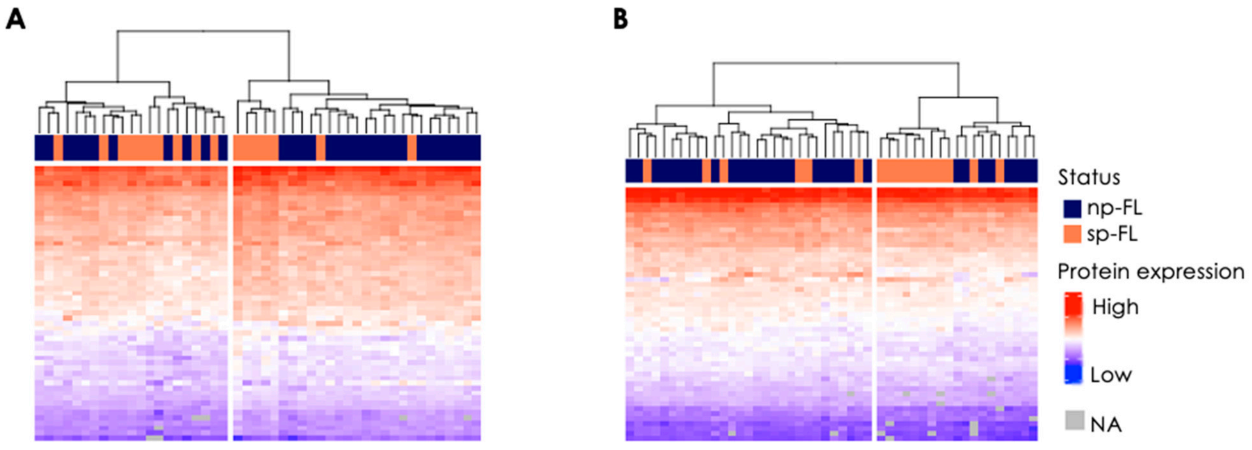

Supplementary Figure S4. Proteins identified from LRG and HRG analysis ability to separate the entire cohort. ***A)*** *55 proteins identified from the low-risk cluster used for hierarchical clustering in the entire cohort.* ***B)*** *51 identified proteins identified from the high-risk cluster used for hierarchical clustering in the entire cohort. Abbreviations: NA, not assigned; np-FL, non-progressing follicular lymphoma; sp-FL, subsequently-progressing follicular lymphoma.*

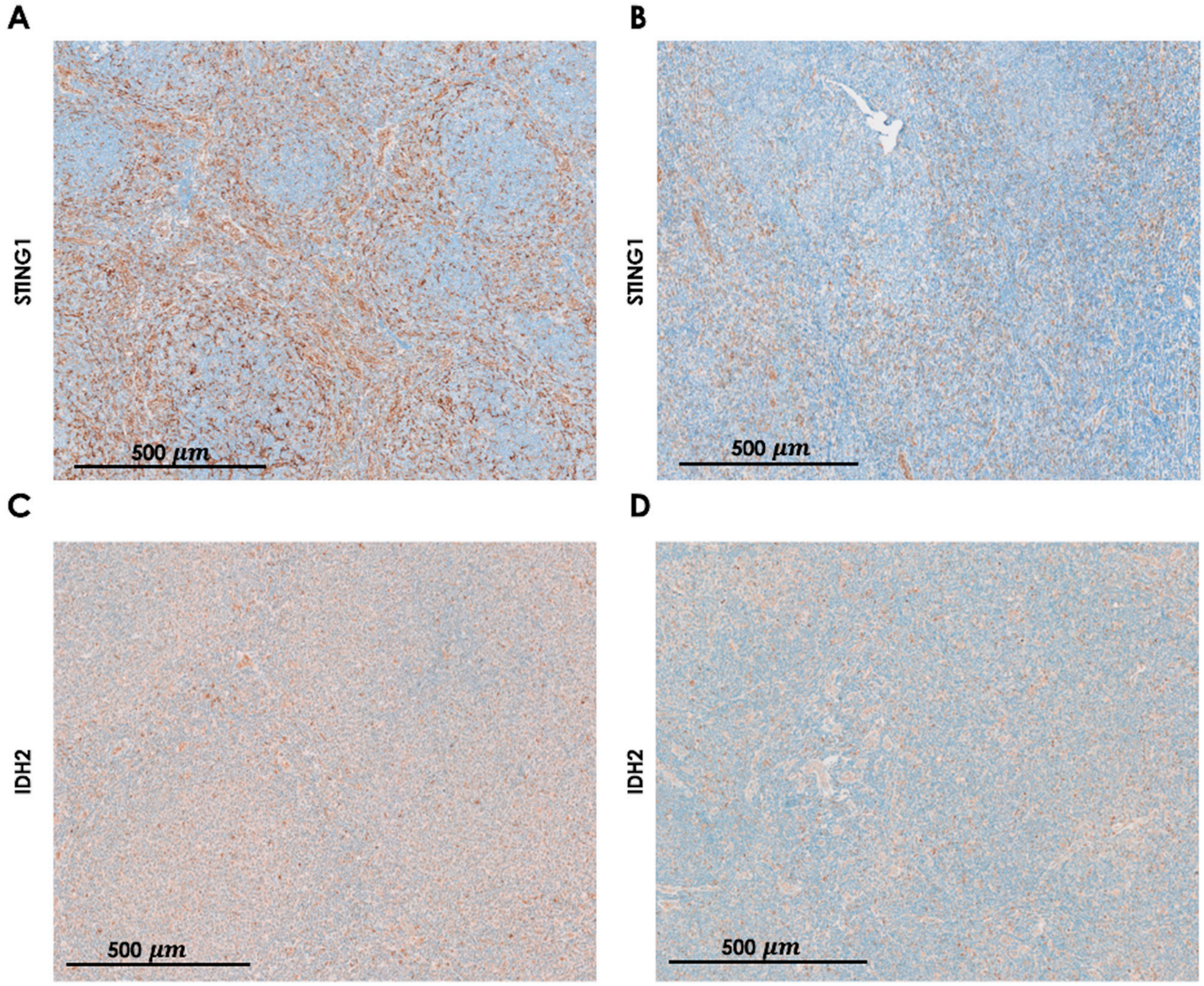

Supplementary Figure S5. Representative images of STING1 and IDH2 staining. ***A)-B)*** *Representative images of STING1 staining. 500 µm line scale bar (5x magnification).* ***C)-D)*** *Representative images of IDH2 staining. 500 µm line scale bar (5x magnification).*
